# In This Issue

**Published:** 2011

## ADVANCES IN ALCOHOLISM TREATMENT

Researchers are working on numerous and varied approaches to improving the accessibility, quality, effectiveness, and cost-effectiveness of treatment for alcohol use disorders. In this overview article by Dr. Robert B. Huebner with Ms. Lori Wolfgang Kantor, the authors summarize these approaches, such as medications development, behavioral therapy, advances in technology that are being used to improve treatment, integrated care of patients with alcohol use disorders and co-occurring disorders, the role of 12-step programs in the broader realm of treatment, treating patients with recurring and chronic alcohol dependence, strategies to close the gap between treatment need and treatment utilization, and how changes in the health care system may affect the delivery of treatment. (pp. 295–299)

## MEDICATIONS FOR UNHEALTHY ALCOHOL USE: ACROSS THE SPECTRUM

The spectrum of unhealthy alcohol use can be addressed in a variety of health care settings, including primary care, specialty practice, and alcohol treatment programs. Medication use in nonspecialized settings and in a spectrum of patients including nondependent individuals is a recent phenomenon. In this article, Drs. Stephanie S. O’Malley and Patrick G. O’Connor discuss the advent of pharmacotherapy and models of counseling appropriate for use in primary care settings as well as in specialty care. By extending the continuum of care to primary care settings, many people who do not currently receive specialty care may have increased access to treatment. In addition, primary care providers may be able to provide continuing care over time. (pp. 300–312)

## BEHAVIORAL THERAPY ACROSS THE SPECTRUM

A wide variety of behavioral approaches can be effective for patients with alcohol and other drug (AOD) use disorders, including coping skills training, relapse prevention and other cognitive–behavioral treatments, contingency management approaches, brief behavioral interventions, behavioral couples and family treatment, facilitated self-change approaches, and aversion therapy. However, choosing the treatment most appropriate for a given patient remains a challenge. In this article, Drs. Katie Witkiewitz and G. Alan Marlatt, review the different types of behavioral treatment currently available and summarize the evidence for their efficacy. In addition, the authors discuss alternative methods of delivery (e.g., computerized or Internet-based approaches) and novel adjuncts to existing treatments (e.g., mindfulness-based approaches) that may appeal particularly to some patients who had not responded to existing therapies. The AOD treatment field might benefit from developing a research agenda seeking to determine how people change and what mechanisms are responsible for change during the course of behavioral treatments. (pp. 313–319)

## THE USE OF EMERGING TECHNOLOGIES IN ALCOHOL TREATMENT

The role of emerging technologies, such as the Internet and text messaging, is becoming more prominent in the treatment of alcohol and drug dependency. This article by Drs. John A. Cunningham, Kypros Kypri, and Jim McCambridge outlines the rationale for using emerging technologies to help problem drinkers and summarizes the types of technologies already being used. It also reviews the research base supporting their use and summarizes selected examples of emerging technologies that have been developed and implemented as stand-alone interventions and as part of other face-to-face interventions. (pp. 320–326)

## AN E-HEALTH SOLUTION FOR PEOPLE WITH ALCOHOL PROBLEMS

Alcohol use disorders and other addictive behaviors are chronically relapsing conditions that in most cases require longer-term treatment. Currently available continuing-care approaches, however, are not widely used for a variety of reasons. Novel information and communication technologies (ICTs) may help make continuing care more accessible and attractive for many clients. According to Dr. David H. Gustafson, Mr. Michael G. Boyle, Dr. Bret R. Shaw, Mr. Andrew Isham, Ms. Fiona McTavish, Ms. Stephanie Richards, Mr. Christopher Schubert, Dr. Michael Levy, and Ms. Kim Johnson, the recent dramatic increase in the availability of smartphones and their capabilities can be employed for continuing-care approaches based on patient self-management. These approaches integrate features specific to cell phones with advanced capabilities in terms of what type of information can be assessed and which services can be provided. A recent review of the literature has demonstrated the potential effectiveness of ICT-based approaches in managing chronic diseases, including AOD use disorders and other addictions. One example of an addiction-relapse prevention system is the Alcohol Comprehensive Health Enhancement Support System (A-CHESS) Program, which provides a wide range of services, including fast access to counselors in crisis situations, triage and feedback to derail the relapse process, social support, access to relevant information, and more. (pp. 327–337)

## INTEGRATING CARE FOR PEOPLE WITH CO-OCCURRING ALCOHOL AND OTHER DRUG, MEDICAL, AND MENTAL HEALTH CONDITIONS

Most people with alcohol and other drug (AOD) use disorders also suffer from mental health and/or medical problems, which complicate treatment and may contribute to poorer outcomes. In many cases, however, treatment services address only one of the co-occurring disorders at a time. In fact, as Ms. Stacy Sterling, Ms. Felicia Chi, and Ms. Agatha Hinman report, finding integrated treatment that addresses all of a patient’s conditions is the exception rather than the rule, although integrated approaches to treatment may improve patient outcomes. Numerous barriers to optimal treatment integration remain, including differences in education and training of providers in the different fields, organizational factors, and existing financing mechanisms. More recently, interest in the integrated treatment approach has been mounting, and many programs are attempting to incorporate integrated models of care. However, there still is debate regarding the optimal degree of integration necessary to ensure that all patients, from those with very severe AOD problems and co-occurring conditions to those with mild or subdiagnostic levels of these conditions receive the care they need. (pp. 338–349)

## THE ROLE OF MUTUAL-HELP GROUPS IN EXTENDING THE FRAMEWORK OF TREATMENT

Alcohol use disorders, especially alcohol dependence, often are chronic conditions that require numerous episodes of care over many years to achieve and maintain full remission, and professional resources alone have struggled to keep these problems in check. In this article, Dr. John F. Kelly and Ms. Julie D. Yeterian discuss the nature and prevalence of mutual-help groups, particularly Alcoholics Anonymous, and review evidence for their effectiveness, cost-effectiveness, and for the mechanisms through which they may exert their effects. The article also provides details about how health care professionals can facilitate their alcohol-dependent patients’ participation in such groups and reviews the evidence for the benefits of doing so. (pp. 350–355)

## TREATING ALCOHOLISM AS A CHRONIC DISEASE: APPROACHES TO LONG-TERM CONTINUING CARE

Alcohol and other drug use disorders are chronic recurrent conditions, and many patients go through numerous cycles of treatment, abstinence, and relapse. Provision of care that extends past the initial intensive therapy may help break this cycle. Currently available continuing-care approaches involve self-help groups, 12-step group counseling, or individual therapy; however, many patients do not complete initial care and therefore never enter continuing-care programs or they drop out early from the continuing-care program. Drs. James R. McKay and Susanne Hiller-Sturmhöfel explore how extended treatment models using alternative treatment approaches might help these patients. These approaches increasingly blur the distinction between initial and continuing care. They also may involve alternative treatment strategies (e.g., telephone-based interventions) and adaptive treatment algorithms that may help attract and retain patients who are not well served by traditional approaches. (pp. 356–370)

## THE RECOVERY SPECTRUM: FROM SELF-CHANGE TO SEEKING TREATMENT

Research has shown that the majority of people with alcohol problems recognize their situation long before seeking treatment. According to Drs. Jalie A. Tucker and Cathy A. Simpson, recent innovations in alcohol-focused interventions are aimed at closing the gap between population need and the actual use of alcohol treatment services, such as screening and brief interventions, guided self-change programs, and telehealth options, which often are targeted and tailored for high-risk groups. Problem drinkers, illicit drug users, and their social networks should be viewed as consumers of services who exercise choice among many available alternatives, including seeking help or continuing substance use in order to close the gap by developing a spectrum of services that matches population need and is sensitive to the preferences of consumers. (pp. 371–379)

## THE COMMUNITY REINFORCEMENT APPROACH: AN UPDATE OF THE EVIDENCE

The Community Reinforcement Approach (CRA) is a comprehensive behavioral treatment package that focuses on the management of substance-related behaviors and other disrupted life areas. The goal of the CRA is to help people discover and adopt a pleasurable and healthy lifestyle that is more rewarding than a lifestyle filled with alcohol or drug use. In this article, Drs. Robert J. Meyers, Hendrik G. Roozen, and Jane Ellen Smith discuss the science behind the CRA and provide an overview of the treatment program. (pp. 380–388)

## HEALTH SERVICES AND FINANCING OF TREATMENT

Financing, payment, and organization and management of alcohol and other drug treatment services are closely intertwined and together determine whether people have access to treatment, how the treatment system is designed, and the quality and cost of treatment services. In this article, Drs. Maureen T. Stewart and Constance M. Horgan discuss how insurance coverage for alcohol treatment services is limited, and many people with substance abuse problems are uninsured. The Federal Parity Law, in combination with national health care reform, can potentially transform delivery of behavioral health services. The law may lead to increased private and public insurance financing for some types of substance abuse treatment. In addition, the parity law is likely to result in changes to the management of alcohol and other drug treatment services under private and public insurance as insurers will have to apply similar processes to medical and behavioral health care. (pp. 389–394)

